# Cloud Computing Image Recognition System Assists the Construction of the Internet of Things Model of Administrative Management Event Parameters

**DOI:** 10.1155/2021/8630256

**Published:** 2021-12-16

**Authors:** Peikun Xie, Enchen Ma, Zaihua Xu

**Affiliations:** ^1^Nanjing Forest Police College, Nanjing, Jiangsu 210046, China; ^2^Southwest Jiaotong University, Chengdu, Sichuan 610031, China

## Abstract

In order to successfully apply the Internet of Things and cloud computing to the administrative management of spatial structures and realize the systematization, digitization, and intelligence of administrative management, this article draws on research experience in related fields and considers the data characteristics and computing tasks of administrative management. The whole cycle of transmission, storage, postprocessing, and visualization is the main line of research, and a cloud computing-based spatial structure administrative management IoT system is constructed. First, by summarizing the application status of the Internet of Things, the general Internet of Things system is summarized into three levels, and combined with the specific work in the spatial structure administrative management, the overall framework of the spatial structure administrative management of the Internet of Things system is proposed, and the functional sublayers are carried out. Secondly, in response to the above problems, through the traditional image recognition system research and practical application investigation, in order to meet the user's requirements for the computing efficiency and recognition accuracy of the image recognition system, an image recognition system in the cloud computing environment is proposed. It proposes a fuzzy evaluation algorithm of health grade hierarchy analysis optimized for the index system and scoring system and a calculation method that uses time series to identify regular outliers. The optical image pixel-level fusion method and the infrared and visible image fusion method based on complementary information are proposed, and the image fusion software is developed. Finally, in order to enable the application layer to use cluster resources to efficiently and intelligently process massive monitoring data containing redundancy, heterogeneity, anomalies, and many other defects, according to the calculation process of each specific task of data preprocessing and postprocessing in the application layer, demonstrations are made one by one. After analysis, it is concluded that vertical storage of data blocks according to different sensor channels is the optimal strategy.

## 1. Introduction

With the increasing popularity of smart sensing and wireless networking technology in the field of structural administration management, large-span spatial structure administration projects involving major public safety have grown rapidly in recent years [1]. Facing the continuous accumulation of massive amounts of heterogeneous monitoring information, with cloud computing as technical support, the establishment of a spatial structure administrative management Internet of Things system to achieve comprehensive perception, intelligent processing, and visual design of monitoring data has become an inevitable requirement for administrative management construction [2]. They study the organic combination of the Internet of Things, cloud computing, and spatial structure administrative management, establish a spatial structure administrative data management cloud platform, give full play to the advantages of cloud computing and the Internet of Things system, and provide technical support for the storage and postprocessing of monitoring data. It is conducive to the accumulation of research forces, reduces the analysis cost of monitoring data, and improves the scale and comprehensive benefits of the administrative management system [3–5].

With the injection of powerful and flexible storage and computing resources of cloud computing, the collaboration of smart hardware and software will become closer. The Internet of Things system will gradually be different from the traditional collection and display system and become a highly information-based organic whole. With the continuous improvement of wireless sensor technology and intelligent control technology, the intelligent, automated, integrated, platform-based, and visualized administrative management of spatial structure has become an inevitable trend in the development of the industry [6]. The Hadoop platform is a parallel programming model and computing framework for processing massive data and is used for parallel computing on large-scale data sets. Grid computing is a technology that has gradually emerged and developed in recent years. It is currently widely used in many research fields such as distributed supercomputing, distributed instrument systems, data-intensive computing, and remote immersion. Using the supercomputing power of grid computing to solve the problem of image recognition is also one of the current research directions. By improving the image recognition algorithm to the nonparallel algorithm, the program is transplanted to the grid computing platform to run, and the distributed image recognition technology is realized [7]. It has a simple structure, can effectively support data-intensive applications, and is very suitable for the development of parallel digital image processing technology. The Internet of Things is an important part of the new generation of information technology and has become the third wave of the information industry after computers, the Internet, and mobile networks [8–10].

Cloud computing platforms and cloud computing network operators are at the core of the entire dynamic administrative management construction process. When an order comes, the corresponding historical data is processed through the cloud computing center, and the optimal node for the corresponding order is quickly selected for the formation of the Internet of Things. Dynamic administrative management responds to demand, so as to achieve the purpose of improving the overall efficiency of dynamic administrative management. The clustering algorithm is introduced at the transport layer to ensure high-efficiency transmission and effectively reduce data loss; at the application layer, data security levels are classified, and data of different security levels are protected to different degrees. In this strategy, the distribution of permissions must go through strict identity verification, which can avoid the security problems caused by illegal users obtaining illegal permissions in the traditional system. In addition, supporting secondary development is an inevitable requirement for the development of the Internet of Things. It is possible to classify the things in the Internet of Things to facilitate the design of users to meet their own needs. Choosing public cloud services as the development support, we establish a spatial structure administrative management cloud computing network and complete the development of the application layer of the Internet of Things system. The platform responds to various needs of users through internally encapsulated application services and realizes real-time reception, dynamic display, and comprehensive management of monitoring information, thereby establishing a complete spatial structure administrative management IoT system.

## 2. Related Work

In recent years, with the rapid development of communication networks and sensor components, automated and intelligent collection of monitoring data has been realized in structural administrative management technology, which also provides feasibility for real-time online analysis. In order to achieve real-time or even premastering of the state changes of structural performance, in addition to the need to improve the monitoring and collection subsystem to ensure accurate collection and transmission of information, it is also necessary to consider the interaction with users and develop a set of data mining, data display, and data storage. The monitoring data management system is integrated with other functions, so as to analyze and process the monitoring information online in real time and present the processing results to the monitoring users to assist the corresponding decision-making.

Kumari et al. [11] proposed a deep belief network model that can use an algorithm called greedy layer-by-layer training strategy for effective training, which quickly aroused people's enthusiasm for cloud computing network research. Subsequently, Sharif et al. [12] proposed that this training method is also applicable to other different types of deep neural networks and can systematically improve the generalization ability of the network model on the test samples. The convolutional cloud computing network is also a large category of cloud computing. Pasquier et al. [13] first introduced the application of the supervised backpropagation algorithm to this multilevel structure of the convolutional cloud computing network. In terms of speech recognition, Anuradha et al. [14] use the DNN-HMM architecture to replace the original GMM-HMM architecture and use the filter bank features of the original Mel domain to replace MFCC. It even dropped by half. In addition, cloud computing has also achieved high-profile results in pedestrian detection and image segmentation and has surpassed human levels in traffic sign classification. For the impulse cloud computing network, starting with the discovery of impulse neurons, Elhoseny et al. [15] proposed the first bionic impulse neuron model. Although its biological nature is very strong, the computational complexity is too high, so there is subsequent evolution of FHN, ML, IZH, IF, and other neuron models, thus greatly reducing the computational complexity. The learning rules for impulse cloud computing networks have gradually evolved from the original Hebb learning rules to STDP and IP mechanisms, thereby improving the learning efficiency of impulse cloud computing networks [16–18].

The classification and recognition accuracy of the image recognition system largely determines the quality of the entire system. Therefore, in image recognition technology, classification and recognition technology should be paid enough attention to. Through the research and improvement of classification and recognition technology, the computational efficiency and recognition accuracy of image recognition can be improved, and the foundation for wider applications can be laid [19]. At present, the main classification techniques include decision tree induction, Bayesian classification, KNN classification, and artificial cloud computing network. Among these methods, KNN classification is a simple, effective, nonparametric method and has been widely used in text classification, pattern recognition, image and spatial classification, and other fields. The KNN algorithm is a lazy learning method, where the learning program will construct a model until the last moment before classifying the given test set. In classification, the computational cost of this learning method and the need for large storage cost were needed [20, 21].

## 3. System-Assisted Administrative Management Event Parameter IoT Model Construction Based on Cloud Computing Image Recognition

### 3.1. Cloud Computing Hierarchical Architecture

**Figure 1:**
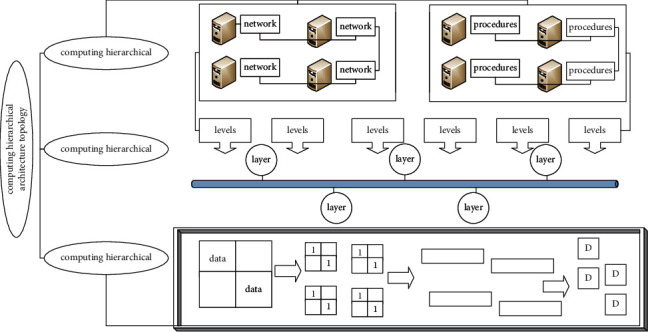
Cloud computing hierarchical architecture topology.

Various services of the cloud computing system exist in the network, users do not need complicated procedures, they only need to access system resources through the Internet, and the cloud computing system supports various access methods, allowing users to access various clients. The information transmission between the three levels of the Internet of Things system is not simple one-way transportation, but a two-way linkage intelligently controlled through a preset real-time feedback mechanism, which involves security technology, analysis and identification technology, service quality management, and cross-level public technology including network management [22].  Figure 1 is the cloud computing hierarchical architecture topology.

Cloud computing is a resource utilization mode, which can access configurable computer resource pools (such as networks, servers, storage, applications, and services) through the network in a convenient, friendly, and on-demand access manner. In this mode, we can quickly supply and provide services with minimal management costs. The main purpose of integrating physical resources is to integrate the services that these physical resources can provide and transform them into resource pools that provide users with different services, including computing resource pools, storage resource pools, network resource pools, and data resource pools.

(1) $$\begin{array}{c} L\times 1+\frac{1}{L+d+N}\approx 1-\frac{1}{L+d+N}. \end{array}$$

The perception layer is the lowest layer in the three-tier system architecture of the Internet of Things, and it contains two application sublayers: data collection and short-distance communication. Its function is to perceive the behavior of the target thing through the sensor node, obtain the information data of the physical world, and then use the self-integrated communication protocol to organize the data exchange and transfer it to the upper layer.

(2) $$\begin{array}{c} M=\left[\begin{array}{cccc} m_{11} & m_{12} & \ldots & m_{1m}\\ m_{21} & m_{22} & \ldots & m_{2m}\\ \ldots & \ldots & \ldots & \ldots \\ m_{m1} & m_{m2} & \ldots & m_{mm} \end{array}\right]. \end{array}$$

The SOA building layer combines the services that cloud computing can provide to users and presents them to users through interfaces, which mainly include service interfaces, service registration, service search, service access, and service workflow. Cloud computing has good interfaces and can realize good interactions with various services.

(3) $$\begin{array}{c} l=\alpha \times \sum_{i=0}^{S^{2}}\sum_{j=0}^{B}\left[{\left(x_{i}-\bar{x}\right)}^{2}+{\left(y_{i}-\bar{y}\right)}^{2}+{\left(x_{j}-\bar{x}\right)}^{2}+{\left(y_{j}-\bar{y}\right)}^{2}\right],\\ L_{\left(\text{emg},k\right)}=\sum_{f\left(\text{emg},k\right)\in A_{\left(\text{emg},k\right)}}\left(f_{\left(\text{emg},n\right)}+C_{\left(\text{emg},k\right)}\right),\\ \min\sum_{i=1}^{N}a_{i}=\frac{1}{2}\sum_{i=1}^{N}\sum_{j}^{N}a_{i}\times y_{j}\times a_{j}\times y_{i}\times k\left(x_{i}^{2},x_{j}^{2}\right). \end{array}$$

As the middle layer of the Internet of Things system architecture, the network layer is mainly used to provide a long-distance communication medium for the two-way information transmission between the perception layer and the application layer on the premise of ensuring the security and integrity of the data. The development of the existing Internet long-distance communication technology has been relatively mature. As long as the data connection with the private network is completed through related protocols, the Internet can safely and in real time transport massive amounts of data in both directions.

### 3.2. Image Recognition Auxiliary Algorithm

From the actual operating point of view, image transformation is to find a suitable orthogonal transformation kernel for the original image. In essence, image transformation has a profound physical background. For example, a Fourier transform of the image reflects the frequency distribution of the function on the system spectrum. Generally, imaging systems only have a certain range of brightness, and the ratio of the maximum value to the minimum value of the brightness becomes the contrast. Using digital image processing technology and pattern recognition theories, combined with computer technology, we analyze various formed elements in urine sediment and find out reasonable segmentation methods and classification rules in accordance with digital image processing algorithms. The system that forms the image has limited brightness, so the contrast is not enough, so the human eye has a poor visual effect when viewing the image. The visual effect is improved through grayscale transformations such as linear transformation, piecewise linear transformation, and nonlinear transformation. Geometric normalization is also called position calibration. It will help correct the size difference and angular deviation caused by the imaging distance and image pose changes. Gray normalization is used to compensate the image obtained under different illumination and light source directions, so as to reduce the change of the image signal caused solely by the illumination change. This step completes the task of finding the image area in the input image. After an image is given, we check whether there is an image in the image, and if so, we give the position and range of each image. Because image detection requires detecting the existence of images and determining their position from various scenes, it is also called image positioning (positioning an image in a scene is also called segmentation). If a static image is an input, each image will be tested. If the input is video, you need to obtain the image area in every frame in the entire sequence. In addition to detecting each frame as a static image, the common strategy is to detect only the first frame of the sequence and track the following frames with the detection results of the previous frame.

**Figure 2:**
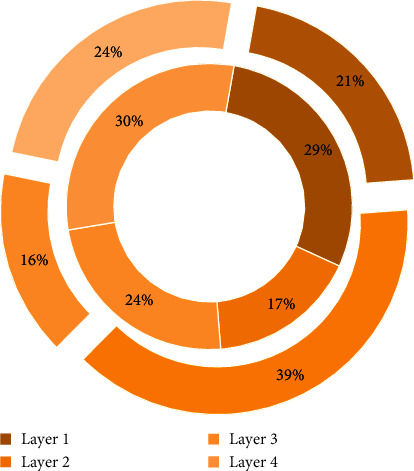
Fan graph of image recognition input information.

Figure 2 is a pie chart of image recognition input information. External input information is transmitted to the cloud computing network through the input layer. The neurons in the input layer transmit information to the hidden layer through a certain relationship. The hidden layer is responsible for information processing. The hidden layer can be set to a single implicit according to data processing needs. After the information is processed, the hidden layer passes the information to the output layer neurons through a certain relationship, and the output layer further processes the data and outputs the information processing results to the outside. The process of inputting external information from the input layer to outputting the output layer is a process of forward transmission and learning of information. If the actual output of the cloud computing network does not meet the expected output requirements, the network will propagate the error back to the output layer, hidden layer, and input layer, layer by layer, and adjust the thresholds of each layer and the adjacent two layers according to the error gradient descent method. The above process is continuously repeated until the output error is reduced to the desired level or reaches the set step. This module is based on the foundation of the subspace image recognition module, provides the function of calculating and storing data, and is the guarantee of the system's operational efficiency. The distributed structure of cloud computing improves the computational efficiency of image recognition algorithms to use HBase to store the image and its feature values for classification services.

### 3.3. Parameter Allocation of Administrative Management Events

Users in the administrative area can obtain the services they need from a complete computer infrastructure through the Internet. IAAS is a business model that allocates hardware resources such as data centers and infrastructure to users through the Web. The advantage of infrastructure services is that users do not need to purchase high-quality equipment but only need to build an application system that meets their needs through Internet leasing. Compared with a single agent, MAS has many unique characteristics: because multiple agents can work in parallel, they can solve the problem very quickly; the communication between the agent and the agent does not need to use raw data but adopts a high-level communication language. This greatly reduces the communication traffic; the function of a single agent is relatively simple, and the agents in the MAS system can cooperate with each other and share tasks to solve some complex large-scale tasks when a problem occurs in a certain agent in the MAS system. Compared with the traditional model, for an enterprise and organization, cloud computing can put the data center in the cloud, and professional companies can provide them with different levels and types of information services. When the responsible task fails, MAS can quickly introduce other agents or replace them with other agents to complete the corresponding tasks, which greatly improves the reliability of the system. Distributed computing technology is also the core of cloud computing technology, which is mainly developed and utilized under the premise of factors such as availability, reliability, and economy. The representative applications of distributed data storage systems in cloud computing mainly include GFS (Google file system) and HDFS (Hadoop distributed file system).

**Figure 3:**
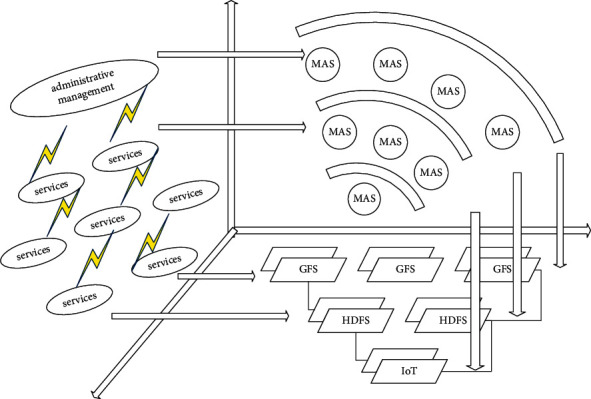
Parameter allocation of administrative management events.

Cloud computing is a comprehensive computing method that combines parallel computing, distributed computing, and grid computing. The development of Internet of Things technology must rely on technologies such as high-efficiency storage and high computing power.  Figure 3 shows the parameter assignment of administrative management events. The Internet of Things combined with cloud computing technology collects and organizes data and information through smart devices such as wireless sensors and radio frequency identification and then transmits it to the cloud computing platform at the application layer to realize data sharing and exchange and complete the control and management of the entire system. By selecting an appropriate matching strategy, the image to be recognized can be matched and compared with the known image in the database, the correlation between them can be established, and the judgment decision made can be output. There are two kinds of recognition purposes and situations that need to be distinguished: one is to verify the image, that is, to confirm whether the person in the input image has an image in the database, which belongs to supervised recognition; the other is to recognize the image, namely, to confirm the identity of the person in the input image, which belongs to unsupervised recognition. Based on the summarized functional requirements analysis, the spatial structure administrative management IoT system should consist of the following parts: a perception network responsible for the collection and control of project site monitoring information and file integration to achieve reliable data between the project site and the remote monitoring and control room transmission network channel according to the user's instructions to call resources to perform various data analyses, and at the same time responsible for the intelligent processing cluster for data storage to provide users with rich visual design and data application display terminals and to realize the user's manual connection with the underlying data through the data interface management terminal for identification and modification.

### 3.4. Weight Update of IoT Model

**Figure 4:**
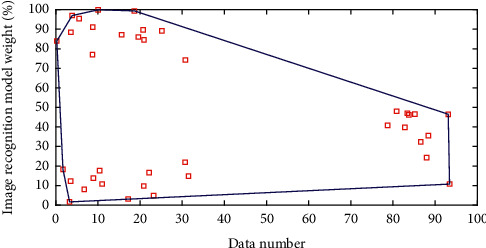
Weight distribution of image recognition model.

Hadoop under the Internet of Things model is a software framework that can perform distributed processing of large amounts of data. It implements Google's MapReduce programming model and framework. It can divide applications into many small work units and put these units in any cluster. In MapReduce, an application that is ready to be submitted for execution is called a “job,” and the unit of work divided from a job that runs and each computing node is called a “task.” In addition, the distributed file system (HDFS) provided by Hadoop is mainly responsible for data storage on each node and achieves high throughput data read and write. In terms of distributed storage and distributed computing, Hadoop uses a master/slave architecture. We run Hadoop through different background programs, which are composed of NameNode, DataNade, JobTracker, and TaskTracker. The NameNode and JobTracker are run on the Master node, and a DataNade and TaskTracker are deployed on each node so that the data processing program running on this node can directly process the data of the machine as much as possible. Other complex issues in parallel programming, such as load balancing, fault-tolerant processing, distributed storage, job scheduling, and network communication, are all handled by the MapReduce framework. The MapReduce processing data set has the following characteristics: the data set to be processed can be decomposed into many small data sets, and each small data set can be processed completely in parallel.  Figure 4 shows the weight distribution of the image recognition model.

The application layer is the top layer in the Internet of Things architecture and the core layer in most Internet of Things systems. The application layer needs to intelligently process and comprehensively manage the collected massive data and provide user-oriented human-computer interaction and data visualization design. The application layer can be divided into an application support sublayer and an application service sublayer. The tasks to be processed by the application support sublayer include postprocessing applications such as data storage, mining, analysis, query, and search. On the basis of data processing, we realize cross-industry, cross-regional, and cross-system information sharing, serving individuals, and organizations in different industries; the application service sublayer is a human-computer interaction interface set up for users to realize the interaction between users and applications order issuance and result feedback. Offline processing in an image recognition system refers to a series of preparations that the image recognition system needs to complete before accepting user queries to recognize images and pictures. This part of the work solves the problem of preprocessing and feature extraction of massive image data, mainly including image and picture preprocessing module, feature value and feature vector extraction module, and feature value access. The main feature of offline processing is that the real-time requirements are not high, but the image and picture index needs to be established in advance.

## 4. Application and Analysis of System-Assisted Administrative Management Event Parameter IoT Model Based on Cloud Computing Image Recognition

### 4.1. Cloud Computing Data Feature Extraction

The network structure of this experiment uses a four-layer deep belief network, the input layer is 784 neurons, the second and third layers as hidden layers each contain 500 neurons, and the last layer is the label layer and contains 10 neurons. We, respectively, represent the network weight between each layer. The Siegert neuron is used for network training, and the training method is the CD algorithm. The experiment content is divided into two parts. The first part is the recognition mode, and the second part is the generation mode. The recognition mode is to give a gray image of handwritten digits to recognize numbers, and the generation mode is to give numbers and let the network generate handwritten digits in reverse. When the feature has a small displacement, the complex cell can ignore this effect, and the information obtained remains unchanged. This feature correspondingly achieves the pooling operation in the convolutional neural network. The grayscale image is reconstructed from handwritten digits. When testing the recognition speed of a single picture in the recognition mode, the LIF neuron is used, and the gray value of the picture needs to be normalized to a value between 0 and 1, and the pulse sequence is determined according to the proportion of each pixel value. In order to further enhance the consistency of the image mode, the statistical characteristics of the sample pictures can be normalized, considering the most basic statistics—the mean value and variance of the grayscale—and adjusting them to a given value.

**Figure 5:**
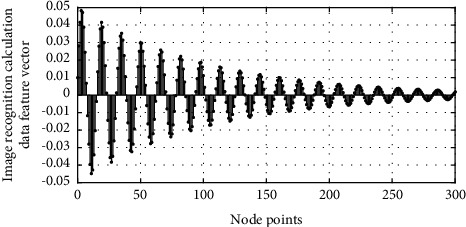
Image recognition calculation data feature vector curve.

Figure 5 is the feature vector curve of image recognition calculation data. When the image boundary is relatively smooth, the area density is smaller, and its density *C* is approximately equal to 1. When the shape of the image area deviates from the circle, the *C* value is smaller. When the edge of the image changes drastically, the perimeter of the image increases, but the area is relatively reduced, resulting in an increase in area density. Therefore, the area density *C* reflects the characteristics of the target topography to some extent. Here, we use edge detection to segment the subimages, because the canny operator has the characteristics of single edge response, accurate positioning, and no false edges. At the same time, it has the first and second derivatives of the image in the area edge extraction advantage. The specific steps are as follows: first, perform median filtering on the subimage extracted in the original image; then, perform image enhancement on the filtered subimage; secondly, use the canny edge detection operator to segment the enhanced subimage. Then, we use the morphological open operation and close operation processing and, finally, remove the small connected domains in the subimage.

### 4.2. Image Recognition IoT System Simulation

Handwritten image recognition is a classic experiment to verify the accuracy and efficiency of the network model. The data set used is the MNIST data set. The training data includes 60,000 handwritten digital grayscale images, and the test set includes 10,000 handwritten digital grayscale images. The image content is a number from 0 to 9, and the pixel size is 28*∗*28. Online processing refers to a series of tasks that the image recognition system needs to complete for the user to feed back the query results after the user enters the image picture or the person's identity information. This part of the work mainly includes the following: according to the image picture that the user needs to query or the person's identity information that needs to be queried, we find the corresponding person's identity information or image pictures from the HBase image database index file. The salient feature of online processing is that the real-time requirements are very high, but all the image and picture data must be compared, so the calculation efficiency is relatively high. In this system, the video capture terminal and the front-end processing terminal are configured in the front-end monitoring, and the monitoring video is collected and obtained through the video capture terminal. The obtained video information can be directly transmitted to the cloud computing system through the transmission network or realize information processing through the front-end processing terminal. Generally, in order to ensure the robustness and usability of the system, multiple video capture terminals from different angles are generally set up in the monitoring area, and the coordination between the terminals is realized through the configuration of monitoring and management software.

**Figure 6:**
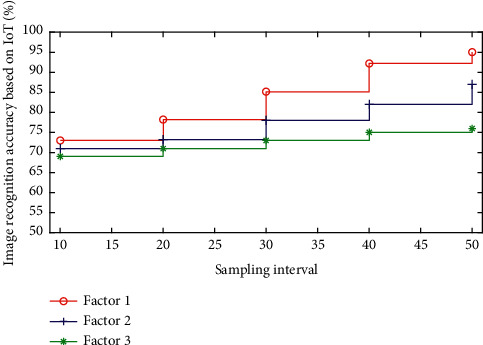
Image recognition accuracy based on the Internet of Things.

In order to port the image recognition application to embedded devices, this section selects the Raspberry Pi 3rd Generation B-type and USB camera as the hardware device. The Raspberry Pi hardware uses the ARM Cortex-A53 chip as the processor, and the operating frequency is 1.2 GHz. Normally, when using a computer to process data, the continuous time signal will be discretized, and the given value is the data at a specific time interval. So, a more realistic assumption is that the sensor will give a measurement result every second. Although the Raspberry Pi is very small, it also has functions such as WIFI and Bluetooth. It can also communicate with other devices through GPIO pins.  Figure 6 shows the accuracy of image recognition based on the Internet of Things. After adding IP, the recognition accuracy of the entire network has improved. It can be clearly seen that 94% is a demarcation point. When the initial discharge rate is greater than 700 Hz, the accuracy fluctuates on the line, but the final value is greater than 94%. In contrast, no IP mechanism was added. When the initial discharge rate was greater than 700 Hz, the recognition accuracy showed a fluctuating downward trend. It can be seen that the time consumed to recognize a single handwritten digital grayscale image is related to the initial discharge rate. After adding IP, when the initial discharge rate is greater than 200 Hz, the average time to recognize a single handwritten digital grayscale image is about 2.5 ms. Without adding IP, when the initial discharge rate is 1000 Hz, the recognition speed is about 5.8 ms. From this analysis, it can be seen that this IP mechanism has improved the recognition accuracy of the network, and the main impact lies in the improvement of the network recognition speed. Then, we want to know why the IP mechanism has such a big impact on the network recognition speed, and it can be seen that when the initial discharge rate is greater than 200 Hz, the broken line tends to be stable, with little fluctuation up and down.

### 4.3. Example Application and Analysis

**Figure 7:**
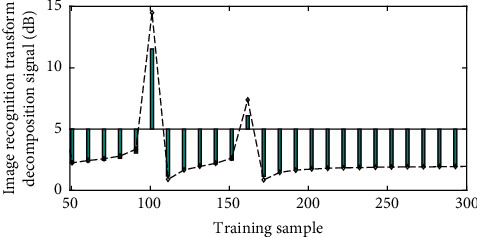
Image recognition transformation and decomposition signal trend.

In the cloud computing network, the ICP adopts a unit modular design and a universal platform that can control and connect a variety of different types of communication systems such as wired and wireless. As the core control device, ICP has dedicated interfaces for different communication methods, and each interface corresponds to a specific communication access device. The ICP interface supports an analog trunking interface, conventional intercom interface, shortwave radio interface, and GSM/CDMA mobile phone interface. Each interface independently completes the receiving and sending of audio signals, and at the same time, it can achieve complete control of the equipment for specific access devices. In order to improve the voice quality, all voice interfaces adopt a four-wire processing method to separate the sending and receiving signals to avoid mutual at the same time; in order to realize that each port can perform two-stage dialing, the external interface in the device has the independent DTMF signal detection and forwarding capabilities.  Figure 7 shows the trend of image recognition transform and decomposing signals.

The process of using wavelet transform to locate the high-frequency components is as follows: decompose the image by wavelet transform, then set the low-frequency part to 0, keep the high-frequency part unchanged, and then perform inverse wavelet transform to complete the localization of the high-frequency components of the original image. In this article, the Mallat algorithm is used to decompose the image, by using Daubechies 9–7 wavelet to obtain 4 subband parts and then zeroing the low-frequency part to ensure that the high-frequency part remains unchanged; then, the reconstruction is performed, and the high-frequency part can be obtained. At the same time, we can select a suitable frequency value, which is between the defocus component, background, and component, and use this frequency value as a threshold for threshold processing. The value above the threshold is 1; otherwise, it is 0; then the place with dense brightness is urine sediment. The position of the components is finally based on the empirical threshold of the image, and the corresponding binary image is obtained by simple discrimination.

**Figure 8:**
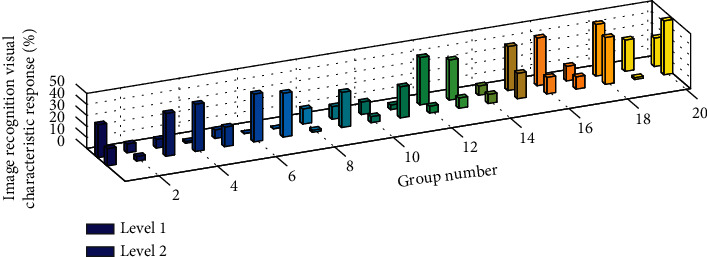
Image recognition visual feature frequency response histogram.

This paper uses the VGG network pretrained on ImageNet as a model for extracting image visual features. The 1 million images provided by ImageNet make VGG can train more accurate features than manual design. In order to make the feature more versatile, this study uses the 4096-dimensional vector output by the layer in VGG-16 as the visual feature of the image.  Figure 8 is a histogram of the frequency response of image recognition visual features. The input layer continuously receives the stimulation of the Poisson pulse sequence over time. When the neuron membrane voltage of the input layer exceeds the threshold, the neuron fires and transmits the pulse to the next layer of neurons. When the third layer of neurons fires, the final result is displayed in the last layer, and the last layer is a 2*∗*5 matrix, which means 0, respectively. The pooling process also has the function of adjusting the output size. In practical applications, for the convolutional neural network model, when the size of the input image is different, in order to obtain a uniform size output, it needs to be realized by pooling operation at this time. The 10 numbers to 9 are numbers, among which the white dots represent the firing neuron and the corresponding identified number. It can be seen that the second neuron is firing, which means that the identified number is 1. When the threshold is unchanged, we change the film time constant between 5 and 20, and the accuracy fluctuates up and down. When the threshold is between 0.2 and 1.8, the accuracy fluctuates greatly. It can be seen that the threshold is one of the main parameters that affect the accuracy of network recognition. When the threshold value is 1.5, the accuracy reaches the highest value of 93.77%.

## 5. Conclusion

This paper proposes a dynamic administrative management construction model based on the cloud computing network, which uses the cloud computing network as the intermediary of the connection and the decision-making body for the construction of administrative management. When choosing, the good classification performance of the evolution cloud computing network is applied, and the resource capacity of the Internet of Things can be selected. According to the matching of information and order requirements, the most suitable node Internet of Things is selected to form an optimal administrative management for the corresponding order. The system uses the traditional image recognition algorithm (PCA) and the cloud computing environment to meet the computing efficiency requirements of users for mass image recognition. Certain improvements take the use of image classification algorithms that assign weights and dynamic *k* values to image images to improve recognition accuracy and stability. Finally, the experiment shows that the system meets the user's requirements for the efficient calculation efficiency and recognition accuracy of the image recognition system. When modeling a dynamic supply chain with time-lag characteristics, applying the orthogonal neural network method can effectively improve the stability of the model, the speed of convergence, and the ability to approximate the optimal solution. The organizational system and structure of dynamic administrative management need to be built on a certain integrated operation platform (including knowledge/skill network, information network, logistics network, and sports network), and the basic platform for dynamic administrative management is also used to a large extent. The above stipulates the specific operation model of dynamic administrative management. Finally, using MatLab to simulate the model, the results show that the dynamic administrative management proposed in this paper has better performance than static administrative management. It can not only reduce costs but also meet more demand orders. The two-dimensional incentive mechanism can effectively stimulate the overall efficiency of administrative management.

## Data Availability

The data used to support the findings of this study are available from the corresponding author upon request.
